# Chronic Administration of a Combination of Six Herbs Inhibits the Progression of Hyperglycemia and Decreases Serum Lipids and Aspartate Amino Transferase Activity in Diabetic Rats

**DOI:** 10.1155/2012/789796

**Published:** 2012-12-09

**Authors:** Reza Shafiee-Nick, Ahmad Ghorbani, Farzaneh Vafaee Bagheri, Hassan Rakhshandeh

**Affiliations:** ^1^Pharmacological Research Center of Medicinal Plants, School of Medicine, Mashhad University of Medical Sciences, Mashhad 9177948564, Iran; ^2^Department of Pharmacology, School of Medicine, Mashhad University of Medical Sciences, Mashhad 9177948564, Iran

## Abstract

The effects of a polyherbal compound, containing six plants (Allium sativum, Cinnamomum zeylanicum, Nigella sativa, Punica granatum, Salvia officinalis and Teucrium polium) were tested on biochemical parameters in streptozotocin-induced diabetic rats. Streptozotocin caused an approximately 3-fold increase in fasting blood sugar level after 2 days. The diabetic control rats showed further increase in blood glucose after 30 days (384 ± 25 mg/dl in day 30 versus 280 ± 12 mg/dl in day 2, *P* < 0.001). Administration of the compound blocked the increase of blood glucose (272 ± 7 and 269 ± 48 mg/dl at day 2 and day 30, respectively). Also, there was significant difference in the level of triglyceride (60 ± 9 versus 158 ± 37 mg/dl, *P* < 0.01), total cholesterol (55 ± 2 versus 97 ± 11 mg/dl, P < 0.01) and aspartate amino transferase activity (75 ± 12 versus 129 ± 18 U/L, *P* < 0.05) between treated rats and diabetic control group. In conclusion, the MSEC inhibited the progression of hyperglycemia and decreased serum lipids and hepatic enzyme activity in diabetic rats. Therefore, it has the potential to be used as a natural product for the management of diabetes.

## 1. Introduction

Diabetes mellitus, a metabolic disease with manifestation of hyperglycemia, is a fast growing health problem through out the world. The World Health Organization estimates that 346 million people suffer from diabetes worldwide. Without urgent action, this number is likely to double by 2030. Generally, diabetes is classified into two main types: type-1 diabetes, a state of insulin deficiency because of defect in islet *β*-cell function and type-2 diabetes which mainly characterized by resistance to the actions of insulin. Over time, diabetes leads to serious microvascular and macrovascular complications such as nephropathy, retinopathy, neuropathy, and cardiovascular disease [1]. Although early onset manifestations of diabetes can be controlled by current oral hypoglycemic drugs or insulin treatment, serious late onset complications appear in many patients [2]. Moreover, the hypoglycemic drugs lead to some unpleasant side effects such as lactic acidosis, peripheral edema, severe hypoglycemia, and abdominal discomfort [3]. Therefore, the search for new antidiabetic agents has continued.

Plants have always been a good source for finding new therapeutic agents for human diseases. Antidiabetic effects of several plants have been supported by results from animal models and clinical trials [4, 5]. Among them, *Allium sativum*, *Cinnamomum zeylanicum*, *Nigella sativa*, *Punica granatum*, *Salvia officinalis,* and *Teucrium polium* are widely used as medicinal plants for management of diabetes in Middle East [6, 7]. Recently, several studies have shown that each one of the six plants is effective in decrease of plasma glucose and serum lipids in diabetes [8–18]. We hypothesized that a combination of their extracts may have more effect on improving metabolic indexes in diabetes. Therefore, the present work was carried out to investigate antidiabetic activity of a polyherbal compound containing these six plant species. 

## 2. Materials and Methods 

### 2.1. Preparation of Extracts

The air-dried *A. sativum* (cloves), *C. zeylanicum* (bark), *N. sativa* (seeds),* P. granatum *(fruits),* S. officinalis* (areal parts), and* T. polium* (areal parts) were powdered and used for extraction. For each plant two types of hydroalcoholic extract were prepared: macerated extract (ME) and Soxhlet extract (SE). The ME was prepared by suspension of each powdered plant material in 70% ethanol and incubated for 72 h at 37°C. The SE was made in a Soxhlet apparatus with 70% ethanol for 24 h [19, 20]. The ME and SE of each plant were evaporated to dryness and then mixed as indicated in Table 1 to make three test compounds: MEs compound (MEC), SEs compound (SEC) and a combination of both MEC and SEC (MSEC).

### 2.2. Animals

Male albino Wistar rats (280–330 g) were used for each experiment. They were housed in a room with controlled lighting (12 h light/12 h darkness) and temperature (22 ± 2°C). The animals were given standard pellets diet and water *ad libitum*. The study protocol using laboratory animals complied with the guidelines of the animal care of the Mashhad University of Medical Sciences, Mashhad, Iran.

### 2.3. Induction of Diabetes

The animals were given a single dose (55 mg/kg, ip) of streptozotocin (STZ) (Enzo Life, USA). Development of diabetes was confirmed by measuring fasting blood sugar (FBS) two days after STZ injection [21, 22]. Rats with FBS level of 250 mg/kg or higher were considered to be diabetic.

### 2.4. Glucose Tolerance Test

Oral glucose tolerance test (GTT) was performed on normal rats that were fasted 16 h. The animals were divided into 4 groups comprising of 8 animals in each groups. The rats in group 1, 2, 3, and 4 were received vehicle, MEC (1 g/kg), SEC (1 g/kg), and glibenclamide (1 mg/kg), respectively. The vehicle or test compounds were given orally thirty minutes before administration of glucose (2 g/kg). A fasting blood sample was first collected from retroorbital sinus, and then three more samples were taken at the 60, 120, and 180 min intervals following glucose administration.

### 2.5. Long-Term Administration of Test Compounds

Diabetic rats were randomized into two groups, 7 animals each: (1) diabetic control rats which were fed standard pellets diet, and (2) diabetic rats which were received diet containing 4% (w/w) of MSEC. The treatment was initiated two days after STZ injection and continued for 4 weeks. At the end of the 30th day, the rats were fasted 16 h and blood samples were collected from retroorbital sinus for biochemical measurements. Also, a 2 h glucose tolerance assay was conducted in the 30th day.

### 2.6. Biochemical Assays

Blood glucose was measured using glucose oxidase reagent (Ziest Chem Diagnostics, Iran). Total cholesterol and high-density lipoprotein (HDL) were evaluated with standard enzymatic colorimetric kits from Pars Azmun (Iran). Serum triglyceride was measured using an enzymatic colorimetric test (Ziest Chem Diagnostics, Iran). Serum alanine aminotransferase (ALT) and aspartate aminotransferase (AST) activities were measured colorimetrically by commercially available kits (Pars Azmun, Iran).

### 2.7. Statistical Analysis

Statistical analysis of changes from baseline was performed by paired *t*-test within groups. Intergroup comparison was done by one-way ANOVA with Turkey's post-hoc test. Results showing *P* values less than 0.05 were considered significant.

## 3. Results

### 3.1. Effect of Test Compounds on Glucose Tolerance

**Figure 1:**
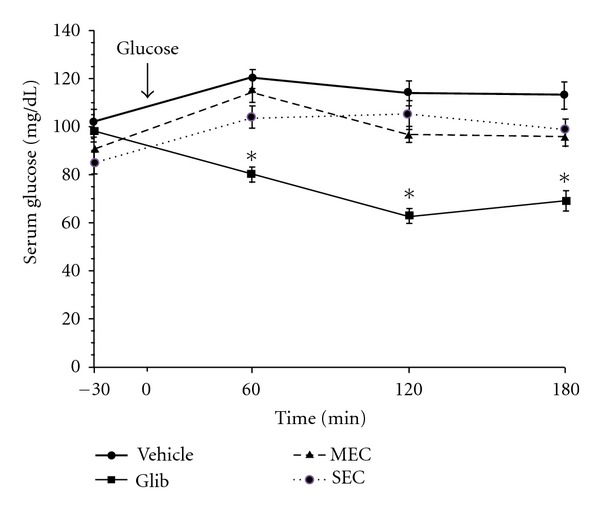
Effect of test compounds on glucose tolerance test (GTT) in normal rats. After 16 h fasting, the animals were received water as vehicle, glibenclamide (1 mg/kg), or test compounds (1 g/kg) orally thirty minutes before administration of glucose (2 g/kg). The data are expressed as mean ± SEM (*n* = 8). *P < 0.01 compared with the corresponding values at all other groups. Glib: glibenclamide-MEC: macerated extracts compound; SEC: Soxhlet extracts compound.

Results of GTT conducted on normal rats are shown in Figure 1. The plasma glucose levels of the control rats reached a peak at 60 min after administration of glucose and gradually decreased. The glibenclamide produced plasma glucose levels significantly (*P* < 0.001) lower than those of the control group at 60–120 min after the glucose administration. When the basal glucose levels were adjusted to 100%, neither the MEC nor the SEC showed significant effect on serum glucose at 60, 120, or 180 min as compared to those of the control group.

### 3.2. Effect of MSEC on Blood Glucose

As shown in Table 2, prior to STZ injection, FBS levels of all the groups were not statistically different from each other. At day 2, administration of STZ to rats caused an approximately 3-fold increase in FBS level compared to normal controls. The diabetic control rats showed further increase in FBS level after 30 days (384 ± 25 mg/dL in day 30 versus 280 ± 12 mg/dL in day 2, *P* < 0.001). However, administration of MSEC to diabetic rats blocked the increase of blood glucose. The level of FBS in this group was 272 ± 7 and 269 ± 48 mg/dL at day 2 and day 30, respectively.

Two hours after feeding of glucose, the blood sugar rose to 451 ± 26 mg/dL from 384 ± 25 mg/dL and to 347 ± 29 mg/dL from 269 ± 48 mg/dL in the case of diabetic controls and MSEC-treated rats, respectively. 

### 3.3. Effect of MSEC on Body Weight and Water Intake

**Figure 2:**
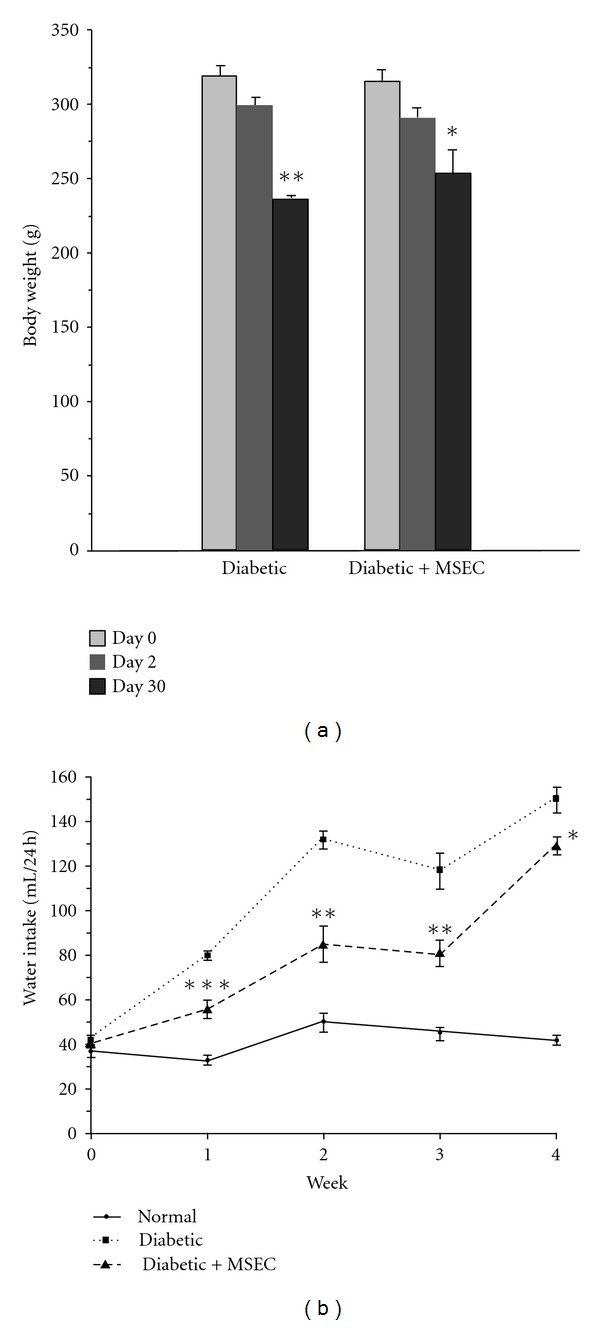
Effect of long-term administration of MSEC on body weight and water intakein diabetic rats. The animals in group of diabetic + MSEC received diet containing 4% (w/w) of MSEC for 1 month. (a): *P < 0.01 compared with the corresponding values at day 2 and day 0. **P < 0.001 compared with the corresponding values at day 2 or day 0. (b): *P < 0.001 and *P* < 0.05 compared with the corresponding values of normal and diabetic + MSEC group, respectively. **P < 0.01 compared with the corresponding values of normal or diabetic + MSEC group. ***P < 0.001 compared with the corresponding values of normal or diabetic + MSEC group. The data are expressed as mean ± SEM for seven (body weight) or six (water intake) rats. MSEC: combination of both macerated and Soxhlet extracts (see Table 1).

After two daysof STZ injection, the diabetic rats in control and MSEC-treated groups showed a significant reduction in their original body weight from 320 ± 6 to 300 ± 5 g (*P* < 0.01) and from 315 ± 7 to 292 ± 6 g (*P* < 0.01), respectively. The weight reduction was continued for both groups and at 30th day reached to 236 ± 4 g (*P* < 0.01 versus day 2) and 254 ± 16 g (*P* < 0.05 versus day 2) for control and MSEC-treated animals, respectively (Figure 2(a)). 

In all groups prior to diabetes induction, the levels of water intake were not significantly different. However, there was a significant increase in the levels of water intake in both groups of diabetic rats after STZ administration (Figure 2(b)). Although the polydipsia condition was evident from the first week to the end of the experiment period, the level of water intake in MSEC-treated rats was significantly lower than that of control diabetic group.

### 3.4. Effect of MSEC on the Levels of Serum Lipids

**Figure 3:**
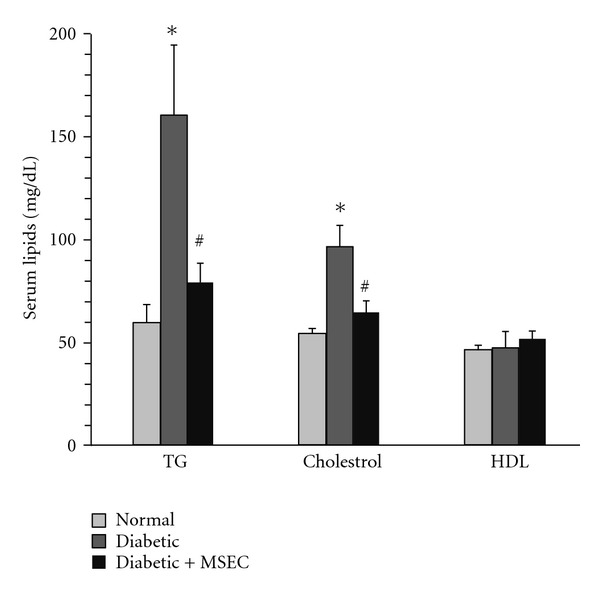
Effect of long-term administration of MSEC on the levels of plasma lipids in diabetic rats. The animals in group of diabetic + MSEC received diet containing 4% (w/w) of MSEC for 1 month. *P < 0.01 versus normal rats. ^#^ *P* < 0.05 versus diabetic group. The data are expressed as mean ± SEM for eight (normal and diabetic + MSEC groups) or six (diabetic groups) rats. MSEC: combination of both macerated and Soxhlet extracts; TG: triglycerides; TC: total cholesterol; HDL: high-density lipoprotein.

Figure 3 shows the effect of MSEC on serum lipids in diabetic rats. There was a significant elevation in the level of triglyceride (60 ± 9 mg/dL versus 158 ± 37 mg/dL, *P* < 0.01) and total cholesterol (55 ± 2 mg/dL versus 97 ± 11 mg/dL, *P* < 0.01) in diabetic rats as compared with normal rats. The MSEC was found to be effective in decreasing the serum lipids. The levels of triglyceride and total cholesterol in MSEC-treated group were 80 ± 9 mg/dL (*P* < 0.01 versus the corresponding value of diabetic rats) and 65 ± 6 mg/dL (*P* < 0.01 versus the corresponding value of diabetic rats), respectively. There was no significant difference between the groups in serum HDL level.

### 3.5. Effect of MSEC on the Serum Enzyme Activity

**Figure 4:**
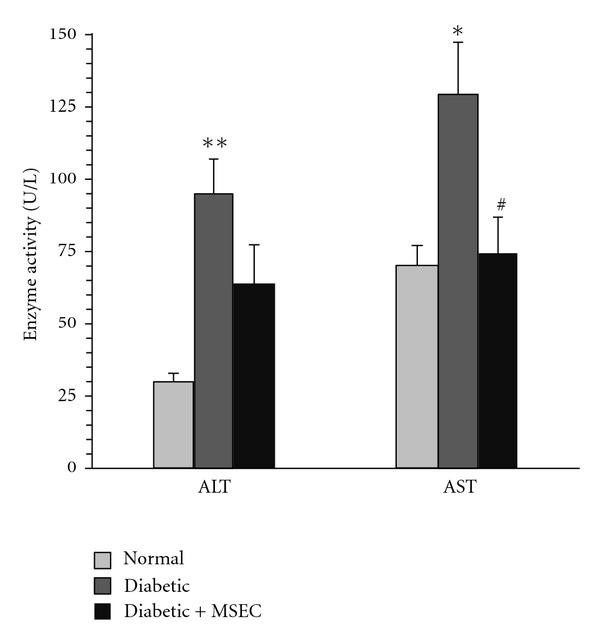
Effect of MSEC on serum alanine aminotransferase (ALT) and aspartate aminotransferase (AST) activities in diabetic rats. The animals in group of diabetic + MSEC received diet containing 4% (w/w) of MSEC for 1 month. *P < 0.05 versus normal and diabetic + MSEC groups. **P < 0.01 versus normal rats. ^#^ *P* < 0.05 versus diabetic group. The data are expressed as mean ± SEM for eight (normal and diabetic + MSEC groups) or six (diabetic groups) rats. MSEC: combination of both macerated and Soxhlet extracts.

After 30 days of diabetes induction, the activity of serum ALT was more than three times relative to the normal animals (95 ± 12 versus 30 ± 3 U/L, *P* < 0.01). Similarly, diabetic rats showed higher AST activity than normal group (129 ± 18 versus 70 ± 7 U/L, *P* < 0.05). Treatment with MSEC decreased the ALT activity (64 ± 13 U/L); however, the effect was statistically insignificant (Figure 4). On the other hand, there was significant difference in the AST activity between MSEC-treated rats (75 ± 12 U/L, *P* < 0.05) and diabetic control group (129 ± 18 U/L).

## 4. Discussion

In the present study, we tested the possible beneficial effects of a polyherbal compound, containing six plants (*A. sativum, C. zeylanicum, N. sativa, P. granatum, S. officinalis, *and* T. polium*) on biochemical parameters of diabetic rats. Although this compound (MSEC) failed to completely restore STZ-induced hyperglycemia and had no remarkable effect on weight reduction; however, it significantly prevented further elevation of blood sugar and improved the polydipsia state. Therefore, it seems that administration of MSEC can only inhibit progression and deterioration of hyperglycemia. Additionally, normal rats treated with MEC or SEC did not change significantly the glycaemia values on GTT, which indicates an antihyperglycemic effect rather than a hypoglycaemic one for the constituents of MSEC. This effect is expected to happen as antihyperglycemic property of its herbal constituents (i.e., the six plants) has been confirmed with repeated studies [8–18].

Antihyperglycemic effect of plants is achieved by enhancing insulin secretion from beta cells, increasing glucose uptake by tissues, decreasing glucose absorption from intestine, inhibiting glucose production in liver, increasing pancreatic tissue regeneration [23], and/or presence of insulin-like agents in plants [4, 23, 24]. Recent studies have shown that *P. granatum* inhibits *α*-glucosidase, rate-limiting enzymes for digestion of oligosaccharides which are necessary for intestinal absorption of glucose. Also, it has been demonstrated that *N. sativa* and *S. officinalis* decrease hepatic glucose production through inhibition of gluconeogenic enzymes. Moreover, beneficial effect of *T. polium* on regeneration of pancreatic islets was reported [25–28].

The levels of serum triglyceride and cholesterol are usually elevated in diabetic patients [29]. The hyperlipidemia mainly occurs as a result of insulin deficiency and thereby dysregulation of metabolic processes like lipolysis and lipogenesis [30]. In the present study also, the diabetic animals showed hypertriglyceridemia, and hypercholesterolemia and the treatment with MSEC significantly decreased the hyperlipidemia. Therefore, the product most probably can prevent dyslipidemia-related complications of diabetic patients. The hypolipidemic action of MSEC is in agreement with earlier studies that reported that *A. sativum*, *C. zeylanicum, N. sativa*, *S. officinalis,* and *T. polium* decrease the levels of serum triglyceride and cholesterol in diabetic animals [8, 11, 12, 16, 18].

Measurements of serum ALT and AST are used in the evaluation of liver damage. Elevation of these enzyme activities is considered as evidence for hepatic damage. An increase of these enzyme activities is also associated with fatty liver disease and decreased hepatic insulin sensitivity in type-2 diabetes [31, 32]. Recently, it has shown that fatty liver disease is associated with an increased risk of death among diabetic patients [33]. In our study, recovery of serum ALT and AST activities of diabetic rats towards normal level shows that the MSEC has protective effect against liver damage and therefore can improve prognosis of diabetic patients. According to the results of previous studies, the main components responsible for this protective effect are most likely found in *A. sativum*, *S. officinalis,* and *T. polium* [8, 16, 34].

In conclusion, the present study demonstrated that MSEC has antidiabetic actions mainly through its hypolipidemic and hepatoprotective effects as well as through inhibition of progression and deterioration of glycemia. Therefore, it has the potential to be used as a new natural product for the management of diabetes.

## Figures and Tables

**Table 1:** Composition of polyherbal compounds.

Herbs	Quantity (g/100 g of compound)		
	MEC	SEC	MSEC (MEC + SEC)
*Allium sativum *	16	16	8 + 8
*Cinnamomum zeylanicum *	14	14	7 + 7
*Nigella sativa *	28	28	14 + 14
*Punica granatum *	14	14	7 + 7
*Salvia officinalis *	14	14	7 + 7
*Teucrium polium *	14	14	7 + 7

MEC: macerated extracts compound; SEC: Soxhlet extracts compound; MSEC: combination of both MEC and SEC.

**Table 2:** Effect of long-term administration of MSEC on blood glucose level in diabetic rats.

Animal groups	Blood glucose (mg/dL)			
	Day 0 (FBS)	Day 2 (FBS)	Day 30 (FBS)	Day 30 (2 h GTT)
Normal control	85 ± 5	82 ± 6	86 ± 8	115 ± 6^#^
Diabetic control	91 ± 5	280 ± 12*	384 ± 25^∗†^	451 ± 26*
Diabetic + MSEC	96 ± 4	272 ± 7*	269 ± 48*	347 ± 29*

The data are expressed as mean ± SEM (*n* = 7). *P < 0.001 compared with the corresponding values at day 0 in each group. ^#^ *P* < 0.05 compared with FBS value at day 30 for normal control group. ^†^ *P* < 0.05 compared with the corresponding values at day 2 for Diabetic control group. FBS: fasting blood sugar; MSEC: combination of both macerated and Soxhlet extracts (see Table 1); GTT: glucose tolerance test.
